# Editorial: Ethical Machine Learning and Artificial Intelligence

**DOI:** 10.3389/fdata.2021.742589

**Published:** 2021-08-12

**Authors:** Novi Quadrianto, Björn W. Schuller, Finnian Rachel Lattimore

**Affiliations:** ^1^ PAL – Predictive Analytics Lab, University of Sussex, Brighton, United Kingdom; ^2^ BCAM Severo Ochoa Strategic Lab on Trustworthy Machine Learning, Bilbao, Spain; ^3^ GLAM – Group on Language, Audio, & Music, Imperial College London, London, United Kingdom; ^4^ EIHW – Chair of Embedded Intelligence for Health Care and Wellbeing, University of Augsburg, Augsburg, Germany; ^5^ Gradient Institute, Sydney, NSW, Australia

**Keywords:** ethics, artificial intelligence, machine learning, fairness, accountability, transparency, trustworthiness, General Data Protection Regulation

## 1 Introduction

Artificial intelligence (AI) and machine learning (ML) have increasingly become an every-day reality for most of us (Elliott, 2019). Typical algorithmic assessment methods, used for predicting human outcomes such as recruitment, bail decisions, mortgage approvals, and insurance premiums, among many others, are currently being trialled and subsequently deployed. Hence, the ethical and legal requirements are moving into the foreground when developing novel AI and machine learning algorithms (Hagendorff, 2020). For example, the United States’ Fair Credit Reporting Act and European Union’s General Data Protection Regulation (GDPR) prescribe that data must be processed in a way that is fair/unbiased—a challenge for AI (Mehrabi et al., 2019). GDPR also alludes to the right of an individual to receive an explanation about decisions made by an automated system such as by explainable AI (XAI) (Gunning et al., 2019).

Here, based on a recent research topic held in Frontiers in Big Data, we provide an overview on the authors’ views and contributions.

This research topic covers but is not limited to the fields of fairness, accountability, transparency, and trustworthiness (Baird et al., 2019), and covers methods such as causality and counterfactual reasoning, reinforcement learning, and probabilistic approaches.

## 2 Literature Review

The research topic provides two overviews on the field.

In the first, Wells and Bednarz discuss in “*Explainable AI and Reinforcement Learning – A Systematic Review of Current Approaches and Trends*” 25 studies selected from 520 search hits on this recent topic. Thereby, they focus on “visualisation, query-based explanations, policy summarisation, human-in-the-loop collaboration, and verification” which they identify as trends. As others, they name the urge for user evaluations including laymen of explanations and find examples often over-simplified going hand-in-hand with lack in scalability, while provision of comprehensible explanations remains a key challenge. Further, they consider more progressive visualisation approaches under-exploited including multimodal and immersive forms of visualisation. Ideally, in the authors’ opinion, such would be combined with “well articulated explanations”.

Next, in their mini review “*Considerations for a More Ethical Approach to Data in AI: On Data Representation and Infrastructure*”, Baird and Schuller observe that data infrastructures are increasingly managed more democratically, as decentralisation fosters transparency and therefore can help better cope with selection-bias. Their review deals with AI-targeted data representation and infrastructures focussing on “auditing, benchmarking, confidence and trust, explainability and interpretability” as key aspects that require attention—ideally also in an interdisciplinary endeavour. As to auditing, in multimodal applications, the authors require standards per modality to lead to accurate benchmarking. Further, they support the view that confidence and trust are benefited by “diverse representations of human data”—the latter also boosting explainability to all users given “inherent human-like attributes”. The authors attest energy put into these aspects by the community, but in particular demand for increased standardisation.

## 3 Technical Approaches

The research topic further includes three technical solutions.

First, in “*The Moral Choice Machine*”, Schramowski et al. demonstrate that one can “extract deontological ethical reasoning” with machine learning from human written texts concerning right or wrong conduct. The authors provide prompts and responses and define a bias score based on the score of positive and negative responses. Likewise, they reach to the Moral Choice Machine (MCM), that determines this score per sentence applying Universal Sentence Encoder embeddings to cater for context. By that, they observe that textual databases bear “recoverable and accurate imprints of our social, ethical and moral choices”. Further, picking selected databases from different epochs, they find reflection on the evolution of these aspects. Similarly, the authors consider different cultural sources. Ultimately, this leads to their view that “moral biases can be extracted, quantified, tracked, and compared across cultures and over time”. As future work, the authors name the possibility to alter the embeddings in targeted ways, such as to eliminate gender stereotypes. They further suggest having the moral choice machine in interactive robots enabled with active learning to have users correct potential biases. Finally, they suggest targeted alteration of the text sources for observation of effects.

In “*Tuning Fairness by Balancing Target Label*s”, Kehrenberg et al. deal with bias in the output as challenge. To this end, they add a latent target output to cater for a unified approach, apply marginalisation rather than constraints problem, and provide for a possibility to integrate knowledge on target unbiased outputs. The authors argue that fairness is usually mainly handled by statistical (group) or individual notions and belief that both are needed for algorithmic fairness. Their approach can be learnt from an implicitly balanced corpus, hence enabling demographic parity and equality of opportunity. They also indicate avenues towards an extension aiming at conditional demographic parity as well. Finally, their general approach uniquely provides for a target rate to control the realisation of the fairness constraint. However, it will need extensions for predictive parity group or individual fairness.

As a third example of algorithmic contribution to a more ethical approach serve Ramanan and Natarajan’s with “*Causal Learning From Predictive Modeling for Observational Data*”. They apply causal Bayesian networks to model causal relationships between data-learnt model variables sequentially using context-specific and mutual independence. Likewise, potential causal relationships are first found. Subsequentially, their strength is determined. The authors verify this approach on benchmark networks and find superiority over current alternatives.

## 4 Discussion

Card and Smith finally discuss “*On Consequentialism and Fairness*”, focusing on the outcome. They argue that consequentialism has its deficits such as lacking in an amenable choice of actions, but is a suited mean to highlight issues in AI fairness such as “who counts”, disadvantages of policy application, or the relative weight of the future. The authors give a consequentialism-based critique of prevailing fairness definitions in AI. They further also take an AI viewpoint on consequentialism. Finally, they elaborate on learning and randomisation in the context of AI ethics.

## 5 Conclusion

As all authors highlight, a more ethical approach is needed to data in AI. However, algorithmic solutions can be and were partially given also here. Accordingly, there is a call to action also for those providing AI algorithms in the first place to actively work on solutions to benefit and protect all users of AI and society.
